# Complete rectal prolapse vs prolapsed hemorrhoids: points to ponder

**DOI:** 10.11604/pamj.2016.24.88.9760

**Published:** 2016-05-27

**Authors:** Susanta Meher

**Affiliations:** 1 Department of Surgery, AIIMS, Bhubaneswar, Odisha State, India

**Keywords:** Rectal prolapse, internal hemorrhoids, radial and circular folds

## Image in medicine

A 45 years female presented with a complain of something coming out through her anus since one year, which comes on straining and reduces only after manual intervention. She had also, a history of constipation with occasional blood and mucus discharge in the stool. On examination, she was found to have full thickness rectal prolapse, which comes out on straining and reduces only after pushing it manually. With a diagnosis of complete rectal prolapse grade III, she underwent abdominal suture rectopexy and now she is doing well after six months of follow-up. One of the very close differential diagnosis of complete rectal prolapse is prolapsed internal hemorrhoid. Both can present with similar symptoms with similar clinical grading, but management is completely different. Diagnosis of both these conditions is critical and mostly based on clinical findings. The differentiating point between a rectal prolapse and internal hemorrhoid lies in the orientation of the mucosal folds. Rectal prolase usually has circular folds (A,B) where as internal hemorrhoids have radial folds (C). This is because, hemorrhoids are collections of submucosal, fibrovascular, arterio-venous sinusoids mostly seen in the left lateral, right anterolateral and right posterolateral region of anal canal. While rectal prolapse is the intussusception of whole circumference of the rectal wall through the anal canal which presents with circular folds of rectal mucosa. This image presents a classic case of complete rectal prolapse with an image of a prolapsed internal hemorrhoid to understand the differentiating points of these two very common clinical conditions.

**Figure 1:**
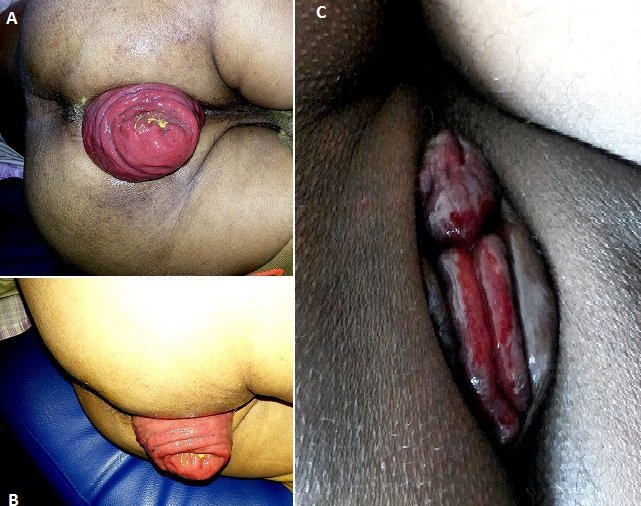
A) complete rectal prolapse with circular folds; B) lateral view of rectal prolapse with circular folds; C) internal hemorrhoids at left lateral, right anterolateral and right posterolateral regions with radial folds

